# 

**Published:** 1879-11

**Authors:** 


					﻿No. 311.
NOVEMBER, 1879.
Price 15 Cts.
TWENTY-SIXTH YEAR.
HALL’S
Journal of Health.!
E. H. GIBBS, A.M., M.D., Editor,
No. 141 EIGHTH STREET (near Broadway) NEW YORK.
Terms, $1.50 a Year, or $1.00 for Eight Months. Postage paid.
Entered as Second-Class matter in the Post-office in New York.
				

